# Stable Solar Water Splitting Enabled in Anodic W/WO_3_ Nanorod Based Electrodes by Hydrothermal Engineering

**DOI:** 10.1021/acsanm.5c03456

**Published:** 2025-09-22

**Authors:** Piyali Chatterjee, Daniel Piecha, Mateusz Szczerba, Olga Chernyayeva, Łukasz Gondek, Tomasz Uchacz, Grzegorz D. Sulka

**Affiliations:** † Faculty of Chemistry, 37799 Jagiellonian University, Gronostajowa 2, 30-387, Krakow, Poland; ‡ Doctoral School of Exact and Natural Sciences, Jagiellonian University, Lojasiewicza 11, 30-348 Krakow, Poland; § Laboratory of Surface Analysis, 119463 Institute of Physical Chemistry, Polish Academy of Sciences, Kasprzaka 44/52, 01-224 Warsaw, Poland; ∥ Faculty of Physics and Applied Computer Science, AGH University of Krakow, Av. Mickiewicza 30, 30-059 Krakow, Poland

**Keywords:** photoelectrochemical water splitting, tungsten oxide nanorods, anodic oxidation, hydrothermal engineering, solar energy conversion

## Abstract

The stability of WO_3_ photoelectrodes in neutral media remains a significant challenge, particularly for those fabricated by anodic W oxidation. We report a simple, one-step hydrothermal treatment that transforms porous anodic WO_3_ into nanorods with a dispersed FeWO_4_ phase. This morphological evolution combines the advantages of high-aspect-ratio structures for improved light absorption with reduced charge recombination losses. The treatment also promotes preferential WO_3_ growth along the monoclinic (002) plane—known to favor water splitting. The modified electrodes exhibited considerable photoluminescence quenching, significantly enhanced charge separation efficiency, and higher photon-to-current conversion, resulting in a photocurrent density that was ∼1.8 times higher at 1.0 V vs RHE. Additionally, oxygen vacancy formation during operation likely contributes to charge redistribution, mitigating surface degradation in sodium sulfate and enabling rapid stabilization of the photocurrent over several hours. Electrochemical impedance spectroscopy reveals evidence of p–n heterojunction due to integration of the tungstate phase with WO_3_, extended charge carrier lifetimes, and enhanced charge transfer. This scalable surface engineering approach offers a promising route to enhance the performance and durability of anodic WO_3_ for practical solar-driven water oxidation.

## Graphical abstract

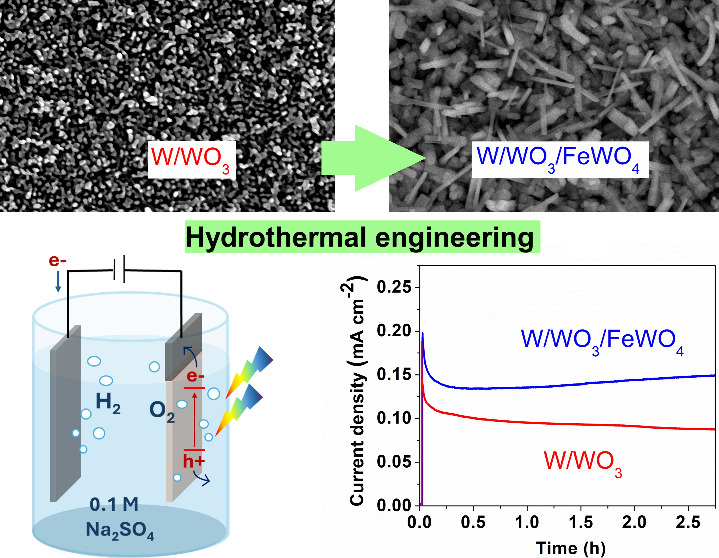

## 1 Introduction

Affordable and clean energy is one of the United Nations’ Sustainable Development Goals to be achieved by 2030.^1^ Sunlight, an abundant and renewable resource, is readily available on Earth, but it is neither continuous nor directly storable.^2^ Therefore, converting solar energy into a portable, carbon-neutral fuel, such as ‘green’ hydrogen, is of paramount importance.^3^ Photoelectrochemical (PEC) water splitting offers a promising route to hydrogen production using light-absorbing semiconductor electrodes, as first demonstrated by Fujishima and Honda in 1972.^4^ This method can significantly reduce external voltage required for electrolysis (well below 1.23 V) by efficient utilization of sunlight.^5,6^ Since water splitting is an energetically uphill reaction, the development of suitable electrode materials remains a major challenge, hindering the commercial viability of this technology. In particular, the oxygen evolution reaction at the photoanode is kinetically sluggish, involving a complex four-electron transfer, and thus has become a key research focus.^7^ Strategies such as heterojunction formation, morphology and phase tuning, incorporation of surface plasmonic features, cation/anion doping, and vacancy engineering are commonly employed to extend the light absorption spectrum, suppress charge carrier recombination and enhance solar energy conversion efficiency of photoelectrodes.^8–14^

Tungsten oxide (WO_3_) is a notable n-type semiconductor with good carrier mobility and excellent stability in acidic media, offering advantages over alternatives such as hematite (Fe_2_O_3_) and titania (TiO_2_).^15–17^ However, several factors prevent WO_3_ from reaching its theoretical performance. Above pH 6, it suffers from severe photocorrosion, and its low absorption coefficient necessitates thick films for adequate light harvesting, which in turn increases bulk recombination of photogenerated electron–hole pairs.^18,19^ To mitigate these issues, electrodes with densely packed morphologies and high aspect ratios are being explored. Such architectures can enhance charge transport by shortening the distance that minority carriers must overcome to reach the electrolyte/semiconductor interface, thereby reducing bulk recombination losses, which are strongly influenced by the carrier diffusion length.^20,21^

Anodic oxidation has been selected in this study among the various scalable and controllable methods for fabricating WO_3_-based electrodes. This technique eliminates the need for post-synthesis coating steps by enabling the direct growth of a nanoporous oxide layer on a W substrate.^22,23^ As a result, the oxide is firmly integrated with the underlying metal, providing robust mechanical stability and minimizing charge losses at the interface with the current collector. The monoclinic crystalline phase of WO_3_ is considered the most efficient for catalytic applications, with enhanced exposure of the (002) crystal plane identified as particularly favorable for water oxidation reaction.^24–26^ In addition to optimizing crystal orientation, it is crucial to mitigate photocorrosion and suppress degradation pathways associated with the accumulation of reactive peroxo- species over time.^27^ WO_3_ can behave as an Arrhenius acid, enabling it to participate in acid–base reactions in alkaline media. Through crystal facet engineering, spatial charge separation can be achieved, extending the applicability of WO_3_ even to photoelectrochemical seawater splitting under alkaline conditions. To improve the long-term operational stability of WO_3_ in practical PEC applications, several surface modification strategies have been reported. These include hydrogen annealing to passivate surface states,^28^ formation of protective amorphous TiO_2_ overlayer by atomic layer deposition (ALD) or electrodeposition,^29,30^ construction of heterojunctions with stable bimetallic oxides,^31^ and deposition of iron oxyhydroxide overlayers that are effective under both mildly acidic (pH 4) and neutral (pH 7) conditions.^32^ Heterojunction formation with tungstates (e.g., CoWO_4_) has also been shown to facilitate interfacial charge redistribution, addressing some limitations of WO_3_ in water oxidation.^33,34^ However, such stabilization approaches have rarely been applied to anodically grown WO_3_. While there are some reports on overlayer deposition, using techniques like SILAR (Successive Ionic Layer Adsorption and Reaction) or spin coating, on nanoporous anodic WO_3_, these studies primarily focus on photocurrent enhancement, with insufficient emphasis on current over time.^35,36^ Overall, to the best of our knowledge, the stability enhancement of anodic WO_3_, particularly in nonacidic electrolytes, remains an underexplored area.^37,38^

In this work, we aimed to address the need for surface protection of anodic WO_3_ through a simple, single-step hydrothermal modification using Fe­(II) precursors in the presence of (WO_4_)^2–^ ions.^39^ This treatment led to the dense growth of WO_3_ nanorods with enhanced expression of the (002) crystal plane, along with the formation of dispersed FeWO_4_. The resulting heterostructure exhibited a significant synergistic effect, contributing to improved PEC stability and a moderate enhancement in photocurrent density.

## 2 Experimental Section

### 2.1 Synthesis of W/WO_3_ and W/WO_3_/FeWO_4_

WO_3_ layers were synthesized on W foil via anodic oxidation, following a previously reported procedure.^40^ The process employed a two-electrode configuration, where a W foil, precleaned with ethanol and acetone, served as the anode, and a platinum wire mesh (with a larger surface area than the anode) acted as the cathode, positioned 2 cm apart. The W foil was cut into 2 × 1 cm^2^ pieces, and insulating paint was applied to restrict the exposed surface area to approximately 0.5 cm^2^. An aqueous electrolyte consisting of 1 M ammonium sulfate ((NH_4_)_2_SO_4_) and 75 mM ammonium fluoride (NH_4_F) was used, maintained at 20 °C under constant stirring. A voltage of 60 V was applied across the electrodes for 60 min. After anodization, the electrodes were thoroughly rinsed with deionized water (three times), then etched in 20% hydrofluoric acid (HF) at 30 °C for 15 s using ultrasonication. This was followed by a brief ethanol rinse lasting 5 s. After removing the insulating paint, the electrodes underwent thermal treatment in air at 500 °C using a muffle furnace, using a heating rate of 2 °C min^–1^ and a total annealing time of 2 h. The samples were then allowed to cool naturally to room temperature and stored for subsequent use. These samples are designated as W/WO_3_.

The W/WO_3_ electrodes were immersed in 35 mL of an aqueous 0.15 mM Mohr’s salt (>99%, Thermo Scientific) solution. Subsequently, 35 mL of aqueous 0.15 mM Na_2_WO_4_·2H_2_O (>99%, Sigma-Aldrich) solution was added dropwise under continuous stirring to initiate the formation of FeWO_4_. Low precursor concentrations were chosen to promote dispersed growth. The mildly acidic pH of the solution minimizes the participation of any WO_3_ dissolution products in the reaction. Stirring was continued for 45 min. Next, two electrodes were arranged in an inclined ‘tent-like’ configuration, with the WO_3_ side facing downward, inside a 100 mL Teflon chamber. The reaction mixture was transferred into the chamber, which was then sealed within a Teflon-lined stainless-steel autoclave and placed in a hot-air oven (without preheating) for hydrothermal treatment at 180 °C for 12 h. After slow cooling to room temperature, the electrodes were removed, rinsed three times with deionized water, and air-dried. Finally, the electrodes were annealed in vacuum at 500 °C for 2 h using a 2 °C min^–1^ heating ramp. These samples, designated as W/WO_3_/FeWO_4_, performed the best during PEC application. A schematic representation of the synthesis process is presented in Figure 1. Another batch was prepared in the same way but with 0.25 mM precursors and these were named W/WO_3_/FeWO_4_ (250).

**Figure 1:**
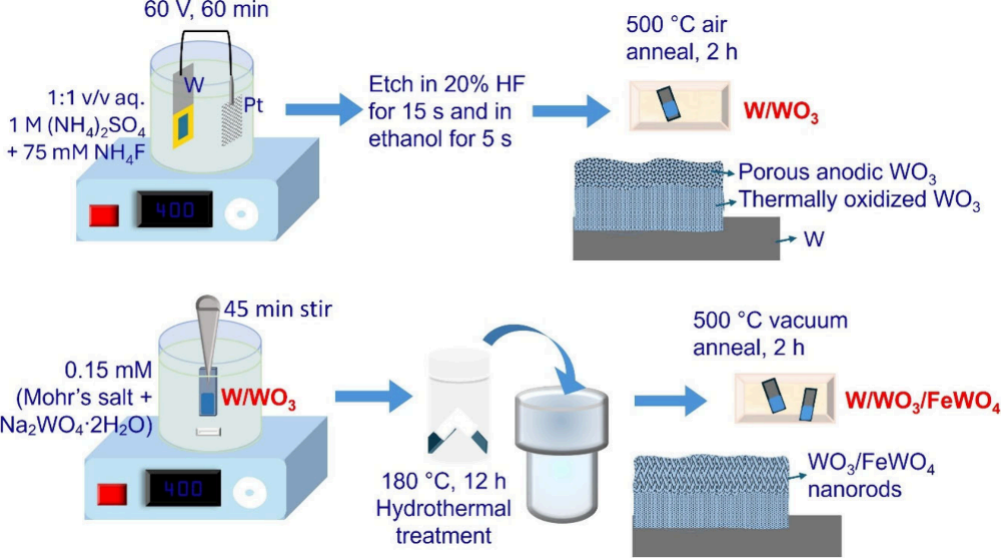
Schematic representation of the synthesis protocol for W/WO_3_ and W/WO_3_/FeWO_4_, along with the corresponding layer composition of the fabricated electrodes.

### 2.2 Characterization Techniques

Surface morphology was examined using a Hitachi S-4700 field emission scanning electron microscope (FESEM), operated at an accelerating voltage of 20 kV and equipped with an energy dispersive X-ray spectroscopy (EDS) system (Thermo Noran System 7) for elemental analysis. To prevent surface charging during imaging at high-voltage imaging, all samples were sputter-coated with a thin gold layer using a current of 10 mA for 120 s. The FESEM images were processed and analyzed using ImageJ software.

For crystallographic analysis (Figure 3(a,b)), X-ray diffraction (XRD) was performed on the electrodes using a glancing-angle Empyrean powder diffractometer by Malvern Panalytical. The X-ray source used was Cu Kα (λ = 1.54 Å). The diffractometer was calibrated for geometry and lines’ profiles by the NIST660 standard sample. The films were aligned at the measuring position using the microscope installed on one of the detector arms. The measurements were carried out in the 10°–123° of 2θ range. Quick scans (Figure 3(c) for FeWO_4_ peak comparison and Figure 6(c) before and after stability tests) were done using a Rigaku Mini Flex diffractometer. Measurements were recorded over a 2θ range of 10–70° with a step size of 0.02°, employing monochromatic Cu Kα radiation generated at an operating voltage of 40 kV.

**Figure 2:**
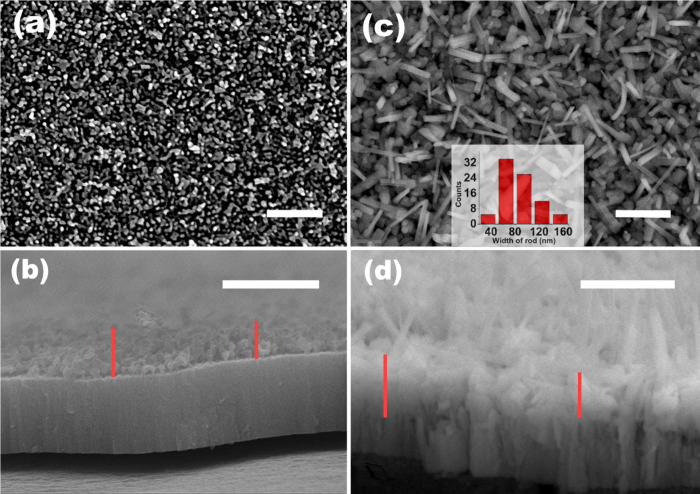
FESEM images of (a) top and (b) cross-sectional views of W/WO_3_. (c) Top view of W/WO_3_/FeWO_4_ with the histogram of nanorod widths (inset), and (d) cross-section of W/WO_3_/FeWO_4_. All scale bars correspond to 1 μm. Red lines indicate the width of the porous anodic layer.

Optical properties were investigated using UV–visible diffuse reflectance spectroscopy (UV–Vis DRS) in the 200–800 nm range, using a PerkinElmer Lambda 750S spectrophotometer. The optical band gap values - either direct (n = 2) or indirect (n = 1/2), were calculated based on the Tauc relation (1):

1 $${\left(Fh\nu \right)}^{n}=A\left(h\nu -E_{g}\right)$$

where *h* denotes Planck’s constant, ν is the photon frequency, *A* represents a proportional constant specific to the material, *E*_*g*_ is the optical band gap energy, and *F*, a function of reflectance (*R*), refers to the Kubelka–Munk function, which serves as an analog to absorbance in diffuse reflectance measurements.^41^

Surface chemical composition and oxidation states were assessed through X-ray photoelectron spectroscopy (XPS), carried out using a Microlab 350 spectrometer equipped with a 20 W Al Kα X-ray source (photon energy: 1486.6 eV). The spectra were analyzed with Thermo Avantage software (version 5.9911), and the C 1s peak at 284.6 eV from adventitious carbon was used for calibration.

Steady state fluorescence spectra were recorded with a Hitachi F-7000 spectrofluorometer and corrected for the spectral sensitivity of the photomultiplier detector. For the steady-state photoluminescence (PL) measurements, excitation was provided by a 340 nm light source. Time-resolved PL measurements were performed with a picosecond Horiba Jobin Yvon spectrofluorometer (UV–Vis–NIR), using a picosecond pulsed UV-LED (λ = 336 nm, pulse width fwhm <1 ns) as the excitation source. During all photophysical measurements, the PL signal was filtered using a 350 nm long-pass filter.

Photoelectrochemical measurements were conducted using a custom-designed Teflon-based three-electrode cell equipped with a quartz window and a PalmSens4 potentiostat. To prevent contact between the tungsten portion of WO_3_ electrodes and electrolyte, the exposed metal was coated with insulating paint. Electrical contact was established at the uncoated top edge, which was connected to the working electrode terminal. A platinum foil and a saturated calomel electrode (SCE) served as the counter and reference electrodes, respectively. The electrolyte consisted of a neutral 0.1 M sodium sulfate solution. Illumination was provided by a solar simulator (Instytut Fotonowy, Poland) using a 150 W xenon arc lamp fitted with an AM 1.5G filter. Linear sweep voltammetry (LSV) was performed at a low scan rate of 5 mV s^–1^ to minimize capacitive dark currents. Measurements were performed under chopped illumination with 5 s light-dark periodic cycles. The applied potential range spanned from just before the photocurrent onset to beyond the water oxidation potential (1.23 V vs RHE, approximately 0.6 V vs SCE). Electrochemical impedance spectroscopy (EIS) was performed using a 10 mV AC perturbation to generate Nyquist plots from frequency sweeps between 0.1 Hz and 100 kHz. Mott–Schottky plots were recorded at a fixed frequency of 500 Hz. To convert the measured potential (*E*) from the saturated calomel electrode (SCE) reference to the reversible hydrogen electrode (RHE) scale, the following Nernst eq 2 was applied:^41^

2 $$E_{\mathrm{RHE}}=E_{\mathrm{SCE}}+0.059\mathrm{pH}+0.219$$

Charge separation and charge injection efficiency measurements were carried out by recording linear voltammograms both in the dark and under simulated solar illumination, with 0.1 M sodium sulfite added to 0.1 M sodium sulfate electrolyte. The charge injection efficiency was calculated as the ratio of the photocurrent density in 0.1 M Na_2_SO_4_ to that obtained in the presence of an additional 0.1 M Na_2_SO_3_ (J_sulfite_). The charge separation efficiency (η_sep_) was determined according to the following relation:

3 $$\eta_{\mathrm{sep}}=J_{\mathrm{sulfite}}/J_{\mathrm{abs}}$$

where, J_abs_ represents the theoretical maximum photocurrent, assuming complete conversion of absorbed photons into current, estimated from absorbance spectra of the electrodes and the spectral intensity of AM 1.5G illumination.^41^

The incident photon-to-current efficiency (IPCE) measurements were carried out using a photoelectric spectrometer (Instytut Fotonowy, Poland). IPCE values were determined from photocurrent densities measured at 1.2 V vs RHE, under monochromatic light with wavelengths increasing in 10 nm steps from 300 to 460 nm. The IPCE% was calculated using the following equation:

4 $$IPCE\%=\frac{1240I\left(\lambda \right)}{P\left(\lambda \right)\lambda }\cdot 100\%$$

where I­(λ) is the photocurrent density (mA cm^–2^), P­(λ) is the incident light power density (mW cm^–2^), and λ is the wavelength (nm).

## 3 Results and Discussion

### 3.1 Material Characterization

The surface morphology was examined using FESEM, and representative images are presented in Figure 2. The top view of the W/WO_3_ electrode (Figure 2(a)) reveals a porous layer composed of walls ∼33–127 nm wide (mean: 61 nm) and channel dimensions ranging from 27 to 131 nm (mean: 63 nm). The corresponding cross-sectional image (Figure 2(b)) shows that the anodically grown porous layer has a thickness of ∼330 nm (up to 531 nm at some regions), beneath which a dense thermal oxide layer is visible. After hydrothermal treatment, a substantial change in morphology is observed in the W/WO_3_/FeWO_4_ sample (Figure 2(c)). High-aspect-ratio nanorods with lengths in the range of ∼334–798 nm (mean: 611 nm) and nanometer-scale widths are formed, as illustrated by the histogram inset. The dense packing of these nanorods is more evident in the cross-sectional view (Figure 2(d)), where the total thickness of the nanostructured layer is ∼495 nm, and reaches up to ∼720 nm. Additionally, the porosity extends into the previously compact thermal oxide layer. These morphological modifications are expected to enhance photoabsorption due to increased surface area and structural depth. Furthermore, the rod-like architecture may help suppress bulk recombination of photogenerated charge carriers by shortening their transport path to the surface. The dense distribution of nanorods also helps limit the exposure of the underlaying tungsten by oxide dissolution during electrochemical operation, potentially improving both performance and stability. However, the nanorods are not vertically aligned, as would have been ideal for absorption and charge transfer in PEC, and instead exhibit a disordered morphology because the source nanostructure (W/WO_3_) itself is not oriented with respect to the substrate. Nevertheless, this disorder may not necessarily be a disadvantage for anodic oxides, since improved coverage of the W substrate reduces its direct exposure to the electrolyte and thereby mitigates corrosion.

A representative EDS spectrum of the W/WO_3_ and W/WO_3_/FeWO_4_ electrodes are presented in Figure S1­(a,b) (Supporting Information). Due to the tungsten substrate and the use of Au sputter coating, quantitative analysis of W:O ratio is not meaningful. As expected, Fe is only weakly detected in the EDS spectrum, consistent with its intentionally low concentration during synthesis and the dominance of the W signal. Notably, no significant contamination from additional precursor elements such as Na or S is observed, indicating successful removal of water-soluble residues during the rinsing steps. However, this observation is not entirely mirrored in the XPS survey scan of the same sample (Figure S2, Supporting Information), which reveals the presence of up to 3.4 at.% of Na, originating from the Na_2_WO_4_ precursor. Interestingly, such Na signal is no longer detected after the photoelectrochemical stability tests, suggesting that sodium ions are gradually leached into electrolyte during operation (detailed discussion in section 3.2).

X-ray diffraction (XRD) analysis was performed to investigate the crystal structures of the electrodes, and a comparison between W/WO_3_ and W/WO_3_/FeWO_4_ is presented in Figure 3. As can be seen in Figure 3(a), both electrodes exhibit high crystallinity, with distinct diffraction peaks corresponding to monoclinic WO_3_ (ICSD #80056)_._ The peaks observed at 40, 58, and 73° can be attributed to the tungsten substrate (ICSD #43667). No significant peak shifts were observed, suggesting that substantial Fe doping into the WO_3_ lattice did not occur. This is likely due the acidic nature of the hydrothermal reaction medium in which WO_3_ is relatively stable. This contrasts with our previous report on hydrothermally engineered anodic WO_3_, where a slightly alkaline environment promoted partial dissolution of WO_3_, enabling Fe ion incorporation into the oxide lattice.^41^ Nevertheless, several low-intensity peaks of monoclinic WO_3_ appear, along with noticeable changes in peak intensity ratios, indicating structural modification of WO_3_ after hydrothermal treatment. As shown in Figure 3(b), a major shift in preferred crystallographic orientation is observed, particularly between the (002) and (020) planes of WO_3_. The (002) plane, which exhibits the highest surface energy, is well-known to enhance water adsorption and photocatalytic oxidation activity, thereby improving the photoelectrochemical potential of W/WO_3_/FeWO_4_.^42^ Additionally, Figure 3(c) displays an intensity expanded view where the most prominent peak of monoclinic FeWO_4_ (ICSD #64733) can be identified at 2θ = 31.7° for both W/WO_3_/FeWO_4_ and W/WO_3_/FeWO_4_ (250), prepared using 0.15 mM and 0.25 mM Fe­(II) precursor solutions, respectively. The relative intensity of this peak increases with precursor concentration. Notably, no diffraction peaks corresponding to Fe_2_O_3_ or Fe_2_WO_6_—compounds containing Fe^3+^—were detected. This absence supports the selection of sodium sulfate as the electrolyte in subsequent PEC studies, given the documented stability of WO_3_ and FeWO_4_ under such conditions, and the known instability of Fe_2_O_3_ in this context.^43^

**Figure 3:**
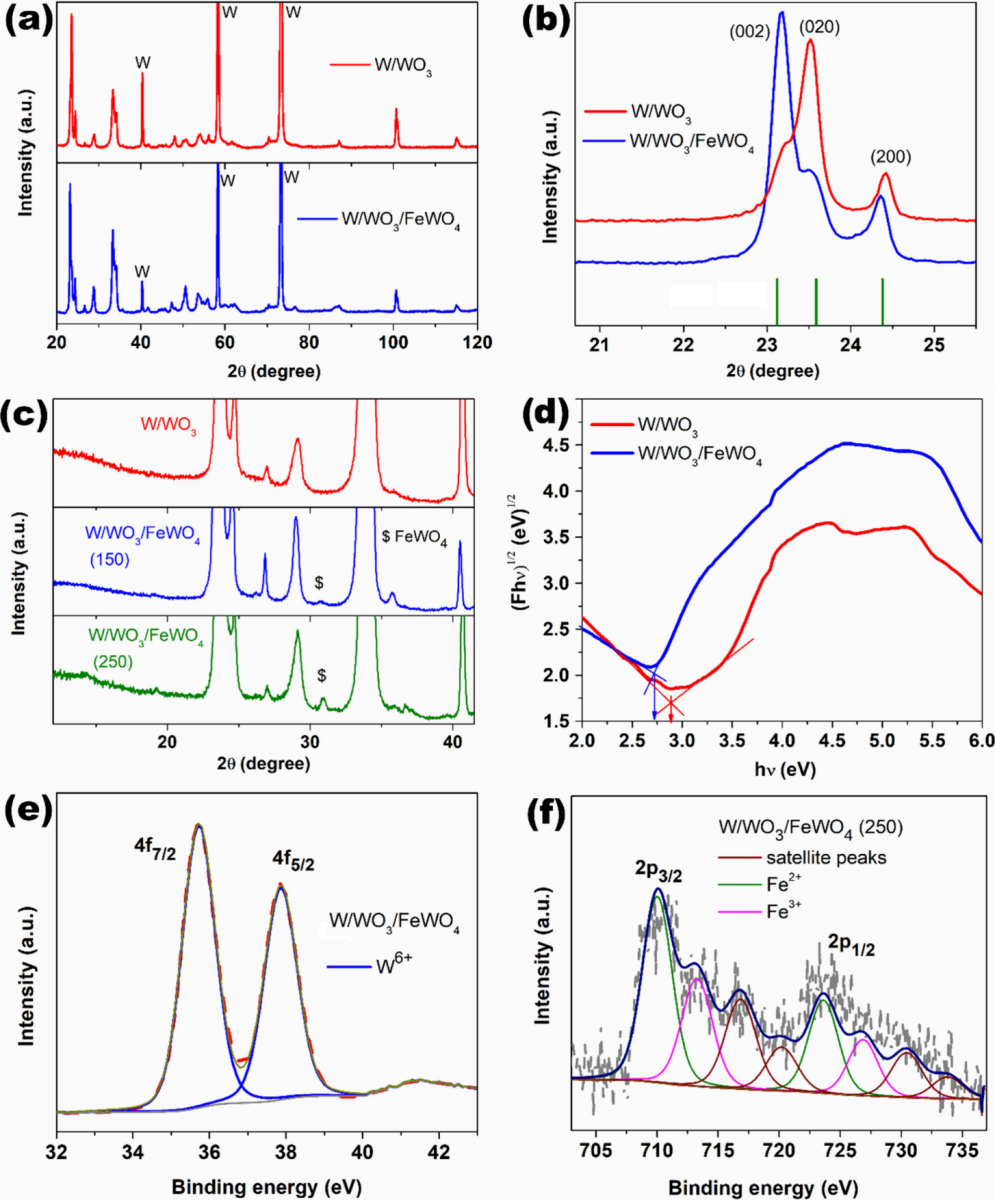
(a, b) XRD patterns of W/WO_3_/FeWO_4_ and W/WO_3_. (c) Truncated XRD patterns of W/WO_3_/FeWO_4_, W/WO_3_/FeWO_4_ (250) and W/WO_3_. (d) Tauc plots derived from UV–Vis DRS spectra for W/WO_3_ and W/WO_3_/FeWO_4._ (e) High-resolution XPS spectrum of W/WO_3_/FeWO_4_ for W 4f. (f) High-resolution XPS spectrum of W/WO_3_/FeWO_4_ (250) for Fe 2p.

UV–Vis DRS measurements was performed for both W/WO_3_/FeWO_4_ and W/WO_3_ electrodes. The recorded data were transformed using the Kubelka–Munk function to obtain absorbance analog values (F), which are dependent on incident photon energy (hν).^44^ These transformed values were used to construct Tauc plots for estimating the indirect optical band gaps of the materials (Figure 3(d)). As shown, W/WO_3_/FeWO_4_ exhibits a band gap of 2.7 eV, compared to 2.9 eV for W/WO_3_ - indicating a band gap narrowing of 0.2 eV. This redshift in the absorption edge is expected to enhance light harvesting and may positively influence PEC performance. The observed band gap reduction is likely due to the surface formation of Fe containing oxides with lower band gaps than WO_3_.^43^

High-resolution XPS spectra of the W 4f region for the W/WO_3_/FeWO_4_ electrode are shown in Figure 3(e). As expected, two prominent peaks corresponding to the 4f_7/2_ and 4f_5/2_ spin–orbit components are observed, separated by 2.1 eV. The deconvoluted spectrum confirms that the dominant oxidation state is W^6+^, indicating that the final thermal annealing step in vacuum effectively minimized surface defect states such as W^5+^. This observation is consistent with the excellent crystallinity revealed by the XRD patterns of both W/WO_3_ and W/WO_3_/FeWO_4_.^45^ The strong presence of W^6+^ further supports the formation of stoichiometric WO_3_, and—as opposed to EDS—signals from metallic W substrate are not expected to interfere in surface-sensitive XPS measurements. It is worth noting that the XPS spectrum of the primary W/WO_3_ electrode also shows a strong W^6+^ signal without any appreciable contribution from W^5+^, as reported in our previous publication on a similar anodic WO_3_ synthesis.^41^ Next, the deconvoluted Fe 2p spectrum of W/WO_3_/FeWO_4_ (250) is presented in Figure 3(f), showing distinct contributions from both Fe^3+^ and Fe^2+^ oxidation states.^46^ The spectrum from W/WO_3_/FeWO_4_ (250) was used for a more reliable estimation of the Fe^2+^:Fe^3+^ ratio, avoiding the low-intensity and noisy Fe signals in the PEC-optimized W/WO_3_/FeWO_4_ sample. The calculated Fe^2+^:Fe^3+^ ratio is 1.66, indicating Fe^2+^-rich surfaces. The presence of Fe^2+^ is consistent with the formation of FeWO_4_, as supported by the XRD data, which identify FeWO_4_ as the primary crystalline Fe-containing phase.

### 3.2 Photoelectrochemical Studies

Linear sweep voltammetry (LSV) under chopped illumination was used to characterize the PEC performance of W/WO_3_/FeWO_4_ compared to W/WO_3._ Due to uncertainties associated with two-step synthesis and possible batch-to-batch variations, two representative samples of each type are presented in Figure 4(a). The observed differences in photocurrent values near the water oxidation potential likely arise from variation in porous layer thickness or the extent of the accessible thermal oxide layer, affecting the width of the space charge region. No conclusive change in onset potential is observed. Nevertheless, W/WO_3_/FeWO_4_ clearly exhibits improved photocurrent density compared to pristine W/WO_3_, with up to 1.8 times higher values at ∼1.0 V vs RHE. Figure S3 (Supporting Information) compares the best LSV curves for W/WO_3_, W/WO_3_/FeWO_4_, and W/WO_3_/FeWO_4_ (250) photoanodes, indicating that increasing Fe precursor concentration, and thus FeWO_4_ content, does not lead to proportional performance enhancement. This is possibly from partial overshadowing of WO_3_ and parasitic light absorption by the added FeWO_4_ layer, a semiconductor with higher absorption edge which may block UV light from reaching WO_3_.^47^ The instantaneous spikes observed in the LSV curves upon light illumination, particularly at lower bias, arise from charge separation within the space charge region. Following photoexcitation, two competing processes, bias-driven electron transfer and electron–hole recombination, result in unstable photocurrent values. Minority carriers (holes, in this case) rapidly accumulate at the surface, and the photocurrent subsequently decays due to recombination with electrons, as charge transfer is comparatively sluggish. At higher bias, however, electron transfer kinetics becomes more favorable for the uphill reaction, enabling even electron–hole pairs generated deeper within the material to overcome spontaneous recombination.^48^

**Figure 4:**
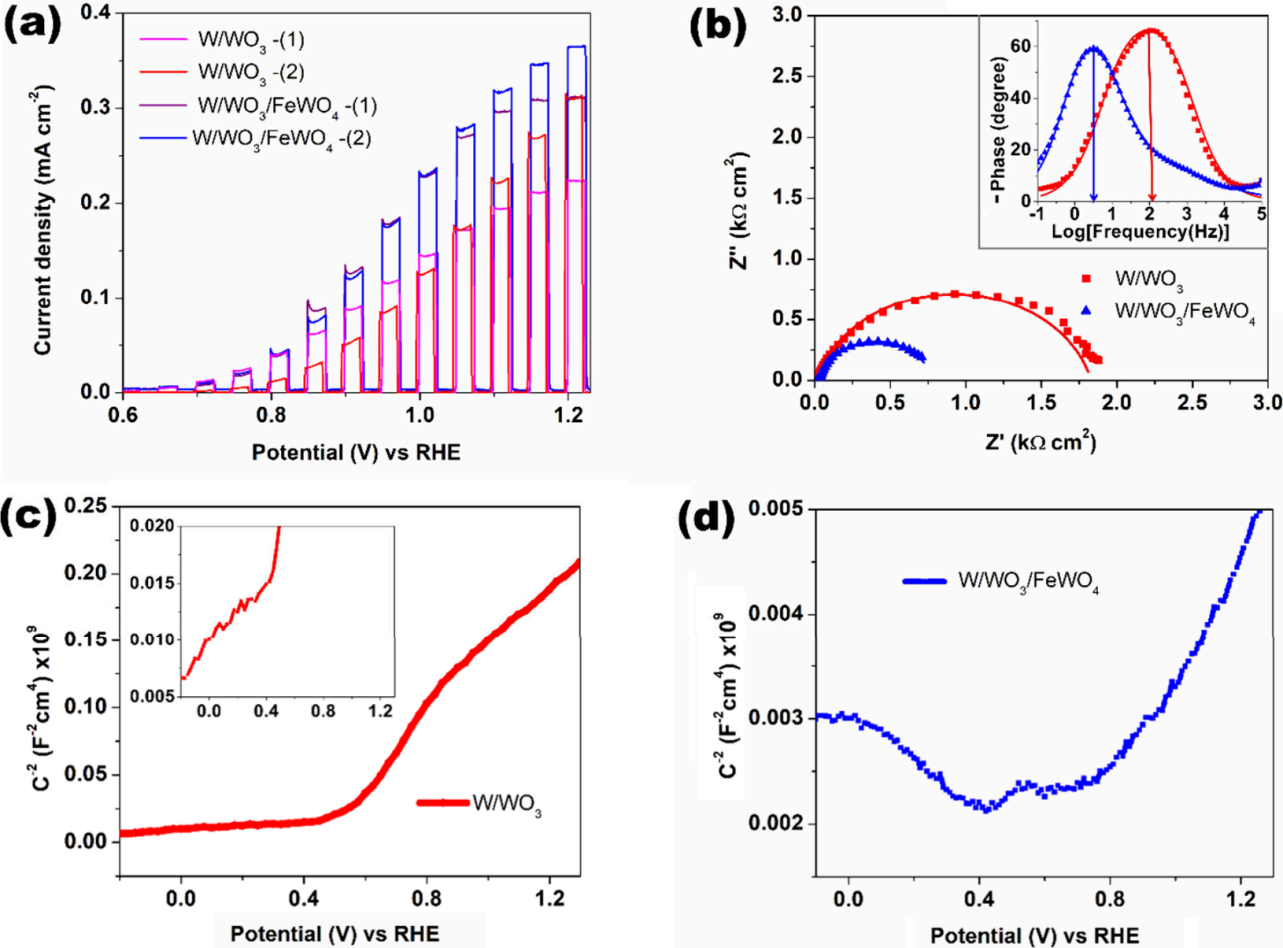
(a) LSV curves of two representative W/WO_3_ and W/WO_3_/FeWO_4_ electrodes in 0.1 M Na_2_SO_4_ under chopped AM 1.5G illumination. (b) Nyquist and Bode phase (inset) plots of W/WO_3_ and W/WO_3_/FeWO_4_ at 1.0 V vs RHE under AM 1.5G illumination; symbols represent experimental data, and solid lines represent the fitted curves. (c, d) Mott–Schottky plots of (c) W/WO_3_ and (d) W/WO_3_/FeWO_4_ recorded at 500 Hz in the dark conditions.

The increase in photocurrent values is further supported by electrochemical impedance spectroscopy (EIS) analysis. Figure 4(b) shows the Nyquist plots (experimental and fitted) of W/WO_3_ and W/WO_3_/FeWO_4_ under illumination at a high bias of 1.0 V vs RHE, conditions under which electron–hole recombination is minimal compared to the rate of charge transfer to water. The corresponding Bode diagrams are provided in Figure S4 (Supporting Information), along with the equivalent circuit models used for fitting the experimental data.^49^ While a simple single RC circuit in series with the solution resistance sufficiently describes W/WO_3_, an additional RC circuit is necessary to account for the surface layer in W/WO_3_/FeWO_4_. This reveals that the diameter of the semicircular arc in the low frequency regime, associated with the charge transfer resistance at the semiconductor/electrolyte interface under illumination, is 2.4 times smaller for W/WO_3_/FeWO_4_ (738.5 Ω cm^2^) than for W/WO_3_ (1813.2 Ω cm^2^). This suggest that W/WO_3_/FeWO_4_ is significantly more effective at facilitating fast electron transfer required for water oxidation. The inset of Figure 4(b) shows the corresponding Bode phase plots. While the peak phase angles are similar, the characteristic frequency (f) is notably shifted, indicating prolonged electron–hole pair lifetime in W/WO_3_/FeWO_4_ (31.8 ms) compared to W/WO_3_ (1.6 ms). The high-aspect-ratio nanorods formed in W/WO_3_/FeWO_4_, as opposed to the porous structure of W/WO_3_, likely enable more efficient transport of photoexcited carriers to the surface for water oxidation.^41,50^ Figure 4(c) and (d) present the Mott–Schottky plots of W/WO_3_ and W/WO_3_/FeWO_4_, respectively. While the positive slope of W/WO_3_ confirms its n-type semiconducting nature, W/WO_3_/FeWO_4_ exhibits both positive and negative slopes, suggesting contributions from both p and n type components^51^—the former possibly arising from FeWO_4_. This dual-type behavior implies the formation of a p–n heterojunction, which can enhance carrier lifetime by providing an internal electric field that drives charge separation and suppresses recombination.^52,53^ It is also worth noting that the C^2–^ values are much lower for W/WO_3_/FeWO_4_, indicating a higher donor density, although the absolute values should not be directly compared due to differences in electrode composition.

To further confirm the superior charge separation properties of W/WO_3_/FeWO_4_, photoluminescence spectroscopy and hole scavenger based tests were performed. The steady-state PL spectra (Figure 5(a)) show significant quenching of PL intensity in W/WO_3_/FeWO_4_, implying that photoexcited electron–hole pairs are more effectively separated and thus less prone to recombination. Time resolved PL measurements were then carried out, with the decay of emission at 580 nm presented in Figure 5(b). The corresponding carrier lifetimes, determined by fitting with a triexponential function, are summarized in Table S1 (Supporting Information). This fitting approach properly captures the slowest decay channel with high overall accuracy. Among the extracted components, the slower decay processes (τ_1_ and τ_2_) are considered more reliable, both due to instrumental sensitivity limitations and their stronger correlation with physical charge carrier dynamics. Notably, τ_1_ and τ_2_ values increase in W/WO_3_/FeWO_4_ at 580 and 460 nm, despite the significantly higher film thickness compared to W/WO_3_. These longer lifetimes indicate that charge carriers can travel greater distances before recombination, thereby increasing their probability of transfer and participating in water redox reactions.^54,55^ Importantly, these measurements represent dry bias-free tests on the electrodes rather than full cell conditions.

**Figure 5:**
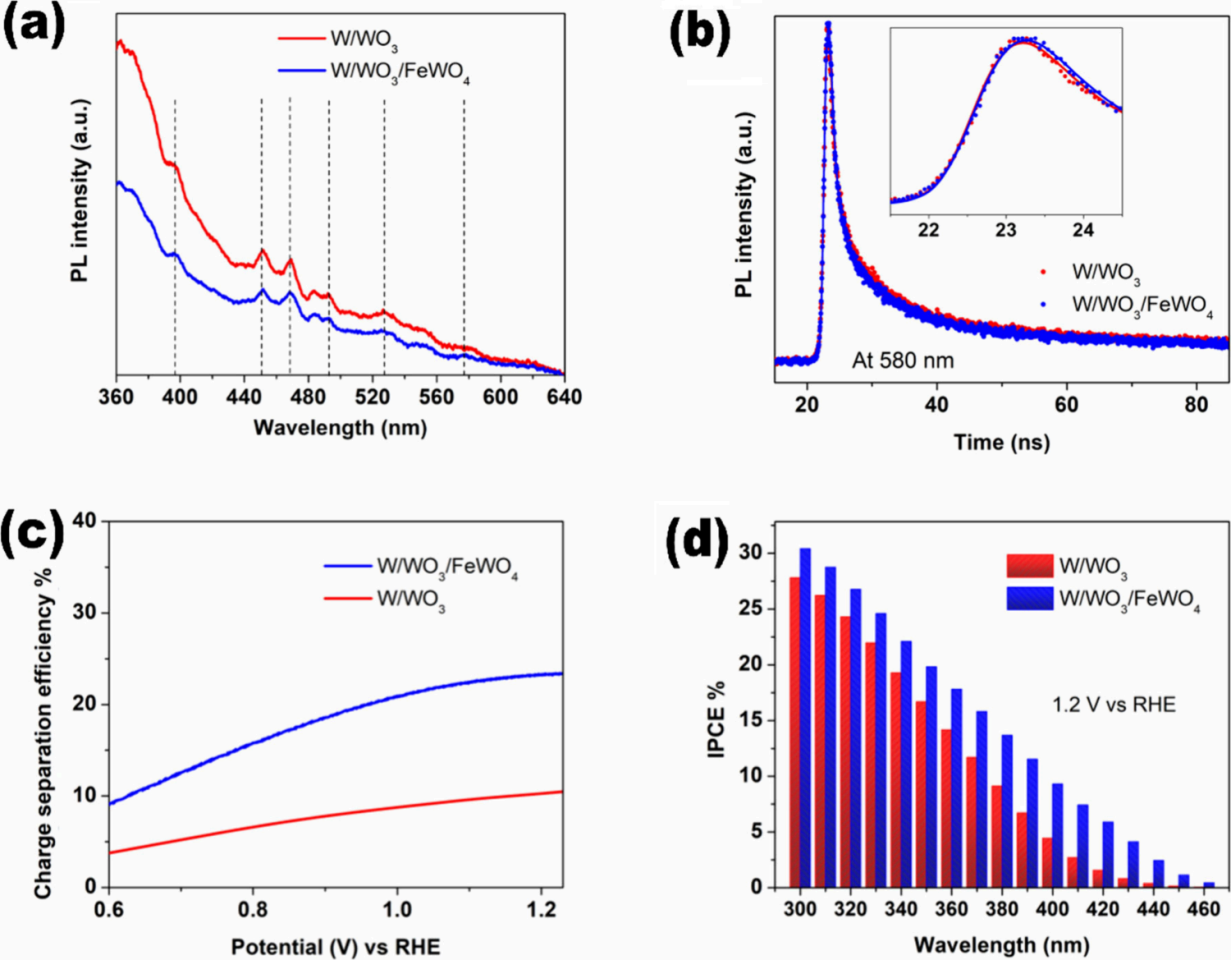
(a) Steady-state PL spectra, (b) time-resolved PL decay at 580 nm, (c) charge separation efficiency, and (d) IPCE of W/WO_3_/FeWO_4_ and W/WO_3_ electrodes.

Charge separation and injection efficiencies in the PEC cell were then evaluated from linear voltammograms recorded with and without 0.1 M sodium sulfite (hole scavenger) in the 0.1 M sodium sulfate electrolyte.^56^ As shown in Figure 5(c), bulk recombination suppression happen and hence charge separation efficiency is markedly improved in W/WO_3_/FeWO_4_. This enhancement can be attributed to the formation of p-n heterojunctions and pronounced (002) crystallographic plane of WO_3_. In contrast, the subsequent step of water oxidation via hole injection was not significantly affected, as evidenced by Figure S5 (Supporting Information), which shows nearly identical charge injection efficiencies for W/WO_3_/FeWO_4_ and W/WO_3_. Although this may seem counterintuitive given the lower R_CT_ values in Nyquist plots, it is likely related to differences in electrode–electrolyte composition and the greater thickness of the W/WO_3_/FeWO_4_ films. Such cases of unchanged C_inj_ despite reduced R_CT_ and extended lifetimes are not uncommon.^57^ A portion of the additional photoexcited carriers generated in the bulk of W/WO_3_/FeWO_4_ is inevitably lost before reaching the surface. This explains why the photocurrent enhancement (∼1.8 times) is lower than the charge separation improvement (∼2.3 times).

Intrigued by the slight decrease in band gap of W/WO_3_/FeWO_4_ compared to W/WO_3_, the IPCE measurements were performed at 1.2 V vs RHE (Figure 5(d)). The results show consistently higher photon-to-current conversion for W/WO_3_ across the entire 300–460 nm range. The enhancement is particularly pronounced in the visible region (400–460 nm), confirming improved photon utilization at the extended light absorption edge. However, since the monochromatic light intensity is low, the photoelectrochemical performances under full -spectrum solar illumination does not directly scale with the integrated IPCE response.

The benefits of modifying W/WO_3_ to W/WO_3_/FeWO_4_ are further demonstrated by photocurrent stability tests at a fixed bias of 1.0 V vs RHE under constant solar-simulated illumination for 3 h. The chronoamperometric curves presented in Figure 6(a) show that while the current slightly decreases over time for W/WO_3_, it remains stable in the case of W/WO_3_/FeWO_4_ after a quick initial drop. The initial decline, caused by the competition between recombination and charge transfer, is better recovered in W/WO_3_/FeWO_4_. The observation is a combined result of its overall better charge separation properties making faster degradation of peroxide species (especially possible at the enhanced (002) plane of WO_3_), changed surface charge distribution^33^ on incorporation of FeWO_4_, and changes in Na^+^ intercalation-deintercalation equilibrium (from Na_2_SO_4_ or precursor Na detected via XPS), which may enable storage/discharge of photoelectrons and creation/healing oxygen vacancies.^58^ According to the literature on WO_3_ photoelectrodes, faster stabilization of photocurrent is a direct indicator of optimized oxygen vacancy incorporation.^59^ When the light is switched off, the current immediately returns to the initial dark level, indicating quick deintercalation and the lack of exposure of W due to WO_3_ photocorrosion. Further characterization of the long-term used electrodes by FESEM (Figure 6(b)) shows no change in morphology after 3 h of continuous PEC testing. The XRD pattern and peak intensity ratios before and after testing (Figure 6(c)) are also nearly identical, indicating no phase changes. This suggests there were no significant areas of exposed W due to dissolution that could have reacted to form new WO_*x*_ phases. Additionally, no peak shift toward lower 2θ values is observed, implying that significant Na^+^ insertion into the lattice did not occur and instead the initial amount was flushed out, as also confirmed by the XPS survey scan in Figure S6 (Supporting Information).^60^ This may explain the slight increase in photocurrent over time, rather than the typical gradual decline. Upon closer surface analysis by XPS, a shift in the W 4f peak toward lower binding energy is observed (Figure 6(d)). Additionally, an increase in intensity is observed, which can be attributed to surface passivation (oxidation) of defects in the anodized electrodes during electrochemical operation in the anodic (positive scan) direction. Moreover, the post-hydrothermal annealing was performed in vacuum, preserving more substrate defects,^61^ which likely undergo complete oxidation during operation. Deconvolution of this spectrum (Figure S7, Supporting Information) reveals a minor presence of W^5+^, which was absent on thesurface of fresh prepared electrodes (compare with Figure 3(e)). The creation of oxygen vacancies during operation, followed by healing through anion adsorption, may enhance the selectivity of the water oxidation reaction (four-hole transfer). Oxygen vacancies particularly enhance the electrochemical water oxidation process in PEC water splitting. Their effect is reflected in reduced overpotential, which translates to increased photocurrent density, rather than a shift in onset potential, as also reported in the literature.^62^ Oxygen vacancies increase conductivity, facilitating bulk charge separation over time. However, unchecked accumulation of oxygen vacancies during operation does not further enhance photocurrent, indicating that defect-healing mechanisms, possibly mediated by FeWO_4_, operate concurrently to minimize trap-assisted recombination in the bulk.^63^ The presence of oxygen vacancies also makes H_2_O_2_ accumulation and consequently WO_3_ dissolution energetically unfavorable, thereby suppressing corrosion pathways.^64,65^ The O 1s spectra of W/WO_3_/FeWO_4_ before and after PEC testing (Figure S8, Supporting Information) show no significant change, indicating that the observed stability is not due to major irreversible surface changes has occurred. However, with mild Ar sputter cleaning, it shows a distinct rise in O vacancies after stability test, as proposed (compare Figure 7(a) and Figure 7(b)). The oxygen vacancies were primarily generated during PEC operation, while the pre-existing vacancies introduced by FeWO_4_ incorporation were much lower (Figure 7(a)) and remain undetectable without surface sputter cleaning (Figure S8, Supporting Information). The oxygen vacancy contribution increased from 15 at.% in fresh samples to 23 at.% after PEC testing, suggesting the beneficial role of surface and subsurface vacancies in the observed performance of W/WO_3_/FeWO_4_.^66^ The improved photocurrent stability of W/WO_3_/FeWO_4_ compared to W/WO_3_ and the extended electrode lifetime suggest better practical utility of anodic WO_3_-based photoelectrodes in neutral electrolytes, which are generally not studied in detail. Despite the moderate enhancement in photocurrent density observed during short illumination periods, the electrodes demonstrate significantly improved performance over extended operation due to complex but beneficial surface chemistry. Changes in crystalline phase composition, such as optimized WO_3_/FeWO_4_ interfacing and enhanced exposure of the (002) plane, along with morphology modifications that promote more directional charge transport, favor PEC water splitting by improving light absorption and charge separation.

**Figure 6:**
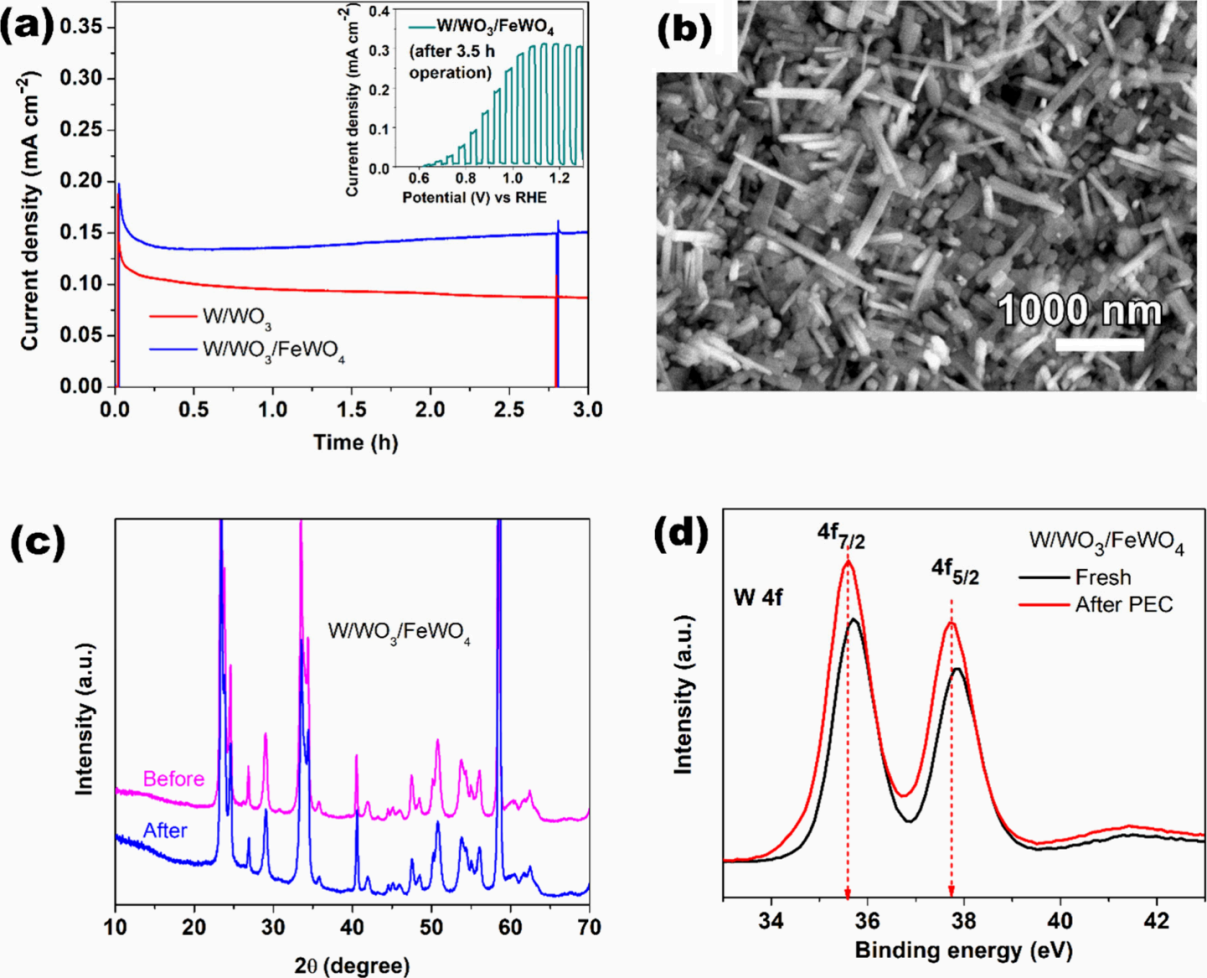
(a) Chronoamperometric curves for the long-time stability test of W/WO_3_ and W/WO_3_/FeWO_4_ electrodes at 1.0 V vs RHE under continuous AM 1.5G illumination for 3 h; (inset) LSV curve of W/WO_3_/FeWO_4_ after the long-time stability test. (b) FESEM image of the W/WO_3_/FeWO_4_ electrode after stability test for 3 h. (c) XRD pattern and (d) high-resolution W 4f XPS spectra of the W/WO_3_/FeWO_4_ electrode before and after the 3 h stability test.

**Figure 7:**
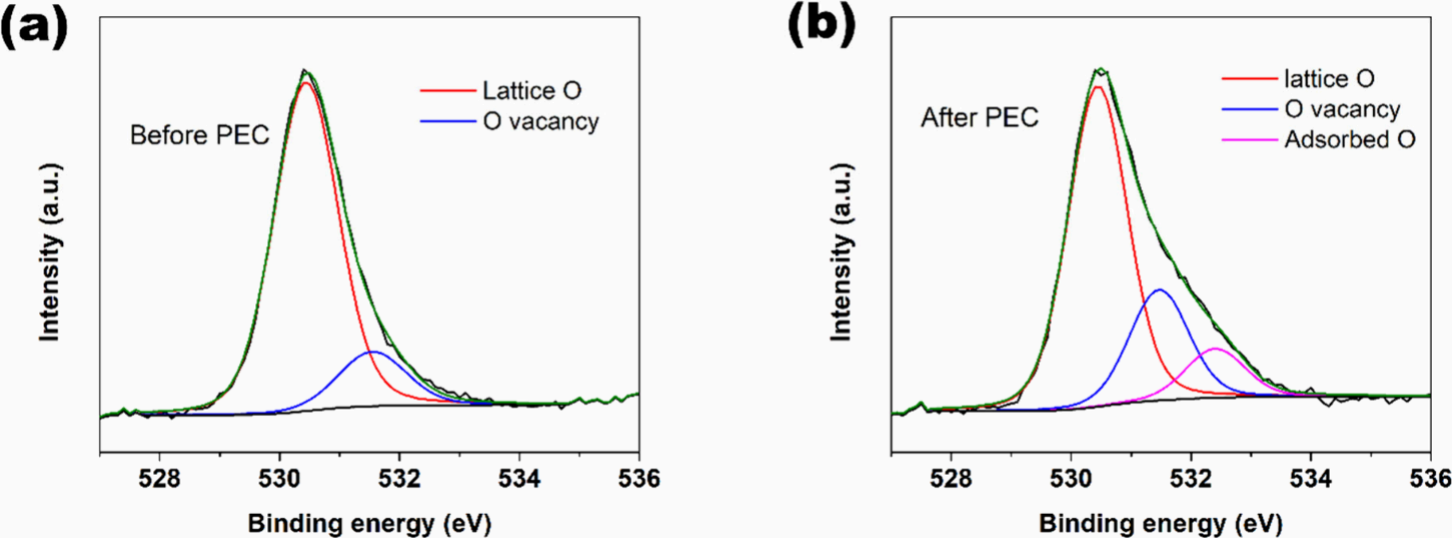
(a) Deconvoluted high-resolution XPS spectrum of W/WO_3_/FeWO_4_ for O 1s after sputter cleaning. (b) Deconvoluted high-resolution XPS spectrum of W/WO_3_/FeWO_4_ for O 1s after the stability test and sputter cleaning.

## 4 Conclusion

In summary, a simple wet chemical approach was demonstrated to significantly improve both the efficiency and long-term operation stability of anodic WO_3_-based photoanodes used for solar water splitting in a sodium sulfate electrolyte. Crystalline n-type monoclinic WO_3_, obtained via anodic oxidation, was subjected to mild hydrothermal treatment in a naturally acidic medium containing highly dilute, equimolar Fe­(II) and W precursors. This process induced a drastic morphology transformation into high aspect-ratio (∼7.5) disordered nanorods, preferential orientation along the favorable (002) WO_3_ plane, a slight reduction in indirect band gap, and the formation of a minor FeWO_4_ phase with nanodiode-like properties. These synergistic modifications led to enhanced photoelectrochemical performance (solar and visible light), marked by photoluminescence quenching, reduced charge transfer resistance, extended carrier lifetimes (electrochemical), and higher charge separation efficiency and photocurrent density. A significant improvement in photocurrent stability under illumination was also observed, likely due to the formation and reversible healing of oxygen vacancies via favorable anion adsorption. This photocurrent stabilization in the neutral electrolyte sets it apart from previous studies that primarily emphasize efficiency enhancement.

## Supplementary Material



## Data Availability

The data presented in this article will be available in RODBUK Cracow Open Research Data repository. https://doi.org/10.57903/UJ/DBCE46.

## Notes

EDS spectra, XPS spectra (before and after stability test), Bode plot, charge injection efficiency plot and photoluminescence decay parameters of W/WO_3_ and W/WO_3_/FeWO_4_; LSV of W/WO_3_/FeWO_4_ (250) compared to W/WO_3_ and W/WO_3_/FeWO_4_. (PDF)

## References

[1] Mohan C., Robinson J., Vodwal L., Kumari N. (2024). Sustainable development
goals for addressing environmental challenges. Elsevier eBooks.

[2] Loh J. Y. Y., Kherani N. P., Ozin G. A. (2021). Persistent
CO_2_ photocatalysis
for solar fuels in the dark. Nat. Sustain..

[3] Choubey P., Rani R., Basu M. (2024). Surface modifications
of a vertically
grown nanostructure for boosting photoelectrochemical water-splitting
performance. ACS Appl. Nano Mater..

[4] Fujishima A., Honda K. (1972). Electrochemical photolysis
of water at a semiconductor electrode. Nature.

[5] Dai Q., Li Y., Qiu Z., Tian H., Pu Y., Chen X., Lv B., Wei J., Wang W. (2024). CdS/ZnFe_2_O_4_ core-shell nanorod
arrays on modified TiO_2_ photoanodes
for photoelectrochemical water splitting. ACS
Appl. Nano Mater..

[6] Wu H., Zhang L., Du A., Irani R., van de Krol R., Abdi F. F., Ng Y. H. (2022). Low-bias
photoelectrochemical water
splitting via mediating trap states and small polaron hopping. Nat. Commun..

[7] Chen Y., Li X., Yang H., Huang Y. (2024). Systematic constructing FeOCl/BiVO_4_ hetero-interfacial
hybrid photoanodes for efficient photoelectrochemical
water splitting. Small.

[8] Liu L., Ruan M., Wang C., Liu Z. (2024). Optimization of the
BiO_8_ polar group of BiVO_4_ by Cl^–^-embedded modification to manipulate bulk-surface carrier separation
for achieving efficient piezo-PEC water oxidation. Appl. Catal. B: Environ. Energy.

[9] Chen X., Ruan M., Wang C., Zhong T., Liu Z. (2024). Phase engineering
to construct In_2_S_3_ heterophase junctions and
abundant active boundaries and surfaces for efficient pyro-PEC performance
in CdS/In_2_S_3_. J. Mater.
Chem. A.

[10] Wang J., Liu K., Liao W., Kang Y., Xiao H., Chen Y., Wang Q., Luo T., Chen J., Li H., Chan T.-S., Chen S., Pensa E., Chai L., Liu F., Jiang L., Liu C., Fu J., Cortés E., Liu M. (2025). Metal vacancies in semiconductor oxides enhance hole mobility for
efficient photoelectrochemical water splitting. Nat. Catal..

[11] Rezaei M., Nezamzadeh-Ejhieh A., Massah A. R. (2025). A Comprehensive
review on the boosted
effects of anion vacancy in the photocatalytic and photoelectrochemical
water-splitting: Focus on oxygen vacancy. Mater.
Today Energy.

[12] Chen P., Kang B., Liu P., Cheng X., Zhong S., Wang X., Fang B. (2025). Passivation
strategies for enhanced
photoelectrochemical water splitting. J. Pow.
Sources.

[13] Li Y., Liu Z., Guo Z., Ruan M., Li X., Liu Y. (2019). Efficient
WO_3_ photoanode modified by Pt layer and plasmonic Ag for
enhanced charge separation and transfer to promote photoelectrochemical
performances. ACS Sustain. Chem. Eng..

[14] Li Y., Liu Z., Ruan M., Guo Z., Li X. (2019). 1D WO_3_ nanorods/2D
WO_3‑*x*
_ nanoflakes homojunction structure
for enhanced charge separation and transfer towards efficient photoelectrochemical
performance. ChemSusChem.

[15] Wu S., Ou K., Zhang W., Ni Y., Tang Y., Xia Y., Wang H. (2024). Fe_2_O_3_/TiO_2_/WO_3_/Ti_3_C_2_T_x_ heterojunction composite material
for efficient photoelectrochemical water splitting. Appl. Phys. A: Mater. Sci. Process..

[16] Dong P., Pan J., Zhang L., Yang X.-L., Xie M.-H., Zhang J. (2024). Regulation
of electron delocalization between flower-like (Co, Ni)-MOF Array
and WO_3_/W photoanode for effective photoelectrochemical
water splitting. Appl. Catal. B Environ. Energy.

[17] Li Y., Liu Z., Li J., Ruan M., Guo Z. (2020). An effective strategy
of constructing a multi-junction structure by integrating a heterojunction
and a homojunction to promote the charge separation and transfer efficiency
of WO_3_. J. Mater. Chem. A.

[18] Ta C. X. M., Furusho Y., Amano F. (2021). Photoelectrochemical
stability of
WO_3_/Mo-doped BiVO_4_ heterojunctions on different
conductive substrates in acidic and neutral media. Appl. Surf. Sci..

[19] Fàbrega C., Murcia-López S., Monllor-Satoca D., Prades J. D., Hernández-Alonso M. D., Penelas G., Morante J. R., Andreu T. (2016). Efficient WO_3_ photoanodes
fabricated by pulsed laser deposition for photoelectrochemical
water splitting with high faradaic efficiency. Appl. Catal. B Environ. Energy.

[20] Liu D., Kuang Y. (2024). Particle-based photoelectrodes for PEC water splitting:
concepts
and perspectives. Adv. Mater..

[21] Feng C., Fu S., Wang W., Zhang Y., Bi Y. (2019). High-crystalline and
high-aspect-ratio hematite nanotube photoanode for efficient solar
water splitting. Appl. Catal. B Environ..

[22] Xia M., Zhao X., Lin C., Pan W., Zhang Y., Guo Z., Leung D. Y. C. (2023). High-voltage
etching-induced terrace-like WO_3_ photoanode for efficient
photoelectrochemical water splitting. ACS Appl.
Energy Mater..

[23] Syrek K., Kotarba S., Zych M., Pisarek M., Uchacz T., Sobańska K., Piȩta Ł, Sulka G. D. (2024). Surface engineering
of anodic WO_3_ layers by in situ doping for light-assisted
water splitting. ACS Appl. Mater. Interfaces.

[24] Ma Q., Song R., Ren F., Wang H., Gao W., Li Z., Li C. (2022). Photoelectrocatalytic
degradation of refractory pollutants
over WO_3_/W network photoelectrode with heterophase junction
for enhancing mass transportation and charge separation. Appl. Catal. B: Environ..

[25] Li H., Shen Q., Zhang H., Gao J., Jia H., Liu X., Li Q., Xue J. (2022). Oxygen vacancy-mediated WO_3_ phase junction to steering photogenerated charge separation for
enhanced water splitting. J. Adv. Ceram..

[26] Alam S., Yamashita H., Verma P. (2025). Unveiling the critical role of high-/low-index
facets in nanostructured energy materials for enhancing the photoelectrochemical
water splitting. ChemCatChem..

[27] Liu J., Xu S.-M., Li Y., Zhang R., Shao M. (2020). Facet engineering
of WO_3_ arrays toward highly efficient and stable photoelectrochemical
hydrogen generation from natural seawater. Appl.
Catal. B Environ. Energy.

[28] Wang G., Ling Y., Wang H., Yang X., Wang C., Zhang J. Z., Li Y. (2012). Hydrogen-treated WO_3_ nanoflakes
show enhanced photostability. Energy Environ.
Sci..

[29] Yang M., He H., Zhang H., Zhong X., Dong F., Ke G., Chen Y., Du J., Zhou Y. (2018). Enhanced photoelectrochemical
water oxidation on WO_3_ nanoflake films by coupling with
amorphous TiO_2_. Electrochim. Acta.

[30] Khan H., Kim M.-J., Baek J.-H., Bera S., Woo H.-J., Moon H.-S., Kwon S.-H. (2022). Sustained
water oxidation with surface-
and interface-engineered WO_3_/BiVO_4_ heterojunction
photoanodes. ACS Appl. Energy Mater..

[31] Loka C., Gelija D., Vattikuti S. V. P., Lee K.-S. (2023). Silver-boosted WO_3_/CuWO_4_ heterojunction thin films for enhanced photoelectrochemical
water splitting efficiency. ACS Sustainable
Chem. Eng..

[32] Lhermitte C. R., Verwer J. G., Bartlett B. M. (2016). Improving the stability
and selectivity
for the oxygen-evolution reaction on semiconducting WO_3_ photoelectrodes with a solid-state FeOOH catalyst. J. Mater. Chem. A.

[33] Zhang H., Tian W., Li Y., Sun H., Tadé M. O., Wang S. (2018). Heterostructured WO_3_@CoWO_4_ bilayer nanosheets
for enhanced visible-light photo, electro and photoelectro-chemical
oxidation of water. J. Mater. Chem. A.

[34] Bai S., Fang Y., Zhao Y., Feng Y., Luo R., Li D., Chen A. (2023). Bi nanoparticles
modified the WO_3_/ZnWO_4_ heterojunction for photoelectrochemical
water splitting. J. Colloid Interface Sci..

[35] Abouelela M. M., Kawamura G., Tan W. K., Matsuda A. (2023). anodic nanoporous WO_3_ modified with Bi_2_S_3_ quantum dots as
a photoanode for photoelectrochemical water splitting. J. Colloid Interface Sci..

[36] Xia L., Bai J., Li J., Zeng Q., Li X., Zhou B. (2016). A highly efficient
BiVO_4_/WO_3_/W heterojunction photoanode for visible-light
responsive dual photoelectrode photocatalytic fuel cell. Appl. Catal. B- environ..

[37] Zhang J., Salles I., Pering S., Cameron P. J., Mattia D., Eslava S. (2017). Nanostructured WO_3_ photoanodes for efficient
water splitting via anodisation in citric acid. RSC Adv..

[38] Fernández-Domene R. M., Roselló-Márquez G., Sánchez-Tovar R., Cifre-Herrando M., García-Antón J. (2021). Synthesis of WO_3_ nanorods through anodization in the presence of citric acid:
formation mechanism, properties and photoelectrocatalytic performance. Surf. Coat. Technol..

[39] Yu F., Cao L., Huang J., Wu J. (2013). Effects of pH on the microstructures
and optical property of FeWO_4_ nanocrystallites prepared
via hydrothermal method. Ceram. Int..

[40] Syrek K., Zych M., Zaraska L., Sulka G. D. (2017). Influence of annealing
conditions on anodic tungsten oxide layers and their photoelectrochemical
activity. Electrochim. Acta.

[41] Chatterjee P., Piecha D., Kotarba S., Syrek K., Pisarek M., Sulka G. D. (2025). Hydrothermal surface
engineering of anodic wo_3_ photoelectrode by simultaneous
iron doping and Fe_3_O_4_/FeWO_4_ formation. ACS Appl.
Mater. Interfaces.

[42] Zhang J., Zhang P., Wang T., Gong J. (2015). Monoclinic WO_3_ nanomultilayers with preferentially exposed
(002) facets for photoelectrochemical
water splitting. Nano Energy.

[43] Chai Q., Dong J., Yu X., Zhang X., Li J., Guo S., Yang Y. (2024). Photocatalytic
activation of oxalic acid over FeOOH
Loaded FeWO_4_/WO_3_ heterojunction for high-efficient
degradation of tetracycline. J. Environ. Chem.
Eng..

[44] Jubu P. R., Obaseki O. S., Ajayi D. I., Danladi E., Chahrour K. M., Muhammad A., Landi S., Igbawua T., Chahul H. F., Yam F. K. (2024). Considerations about the determination of optical bandgap
from diffuse reflectance spectroscopy using the Tauc plot. J. Opt..

[45] Wang Q., Wu H., Wang Y., Li J., Yang Y., Cheng X., Luo Y., An B., Pan X., Xie E. (2021). Ex-situ XPS Analysis
of yolk-shell Sb_2_O_3_/WO_3_ for ultra-fast
acetone resistive sensor. J. Hazard. Mater..

[46] Bagus P. S., Nelin C. J., Brundle C. R., Crist B. V., Lahiri N., Rosso K. M. (2022). Origin of the complex
main and satellite features in
Fe 2p XPS of Fe_2_O_3_. Phys.
Chem. Chem. Phys..

[47] Escaliante L. C., Azevedo F., Mendoza H. E., Xiao C., Kandel R., Humberto J., Osterloh F. E. (2024). Sputter-coated
TiO_2_ films
as passivation and hole transfer layers for improved energy conversion
with solar fuel WO_3_/CuWO_4_ photoanodes. ACS Appl. Mater. Interfaces.

[48] Peter L. M., Walker A. B., Bein T., Hufnagel A. G., Kondofersky I. (2020). Interpretation
of photocurrent transients at semiconductor electrodes: effects of
band-edge unpinning. J. Electroanal. Chem..

[49] Wu W., Yan Z., Wang L., Zhu X., Zhu Y., Liao G., Zhu L. (2024). Efficient WO_3_ nanoplate arrays photoanode modified by
ZnO nanosheets for enhanced charge separation and transfer to promote
photoelectrochemical performances. Adv. Electron.
Mater..

[50] Nikhil S. K., Das A., Kumar P. M., Bhagavathiachari M., Nair R. G. (2021). Effect of aspect
ratio of c-axis oriented ZnO nanorods on photoelectrochemical performance
and photoconversion efficiency. Opt. Mater..

[51] Cao J., Yu H., Zhou S., Qin M., Lau T.-K., Lu X., Zhao N., Wong C.-P. (2017). Low-temperature
solution-processed
NiO_x_ Films for air-stable perovskite solar cells. J. Mater. Chem. A.

[52] Fathabadi M., Zhao S. (2023). Abnormal photocurrent
in semiconductor p-n heterojunctions: toward
multifunctional photoelectrochemical-type photonic devices and beyond. Adv. Electron. Mater..

[53] Hedayati M., Fouladvand M., Rouhollahi A. (2024). Fabrication of WO_3_/Co_3_O_4_ nanorods as a p-n heterojunction photoanode
for efficient photoelectrochemical oxygen evolution. Int. J. Hydrog. Energy.

[54] Wang J., Liao W., Tan Y., Henrotte O., Kang Y., Liu K., Fu J., Lin Z., Chai L., Cortes E., Liu M. (2025). Transfer dynamics of
photo-generated carriers in catalysis. Chem.
Soc. Rev..

[55] Kar A., Majumdar S., Chowdhury P. K., Ganguli A. K. (2025). Unravelling charge
carrier dynamics in Bi_2_MoO_6_/CaFe_2_O_4_ heterostructure for efficient photoelectrochemical
applications. J. Phys. Chem. C.

[56] Meng L., He J., Li S., Zhou X., Xin R., Xu W., Shao B., Tunmee S., Kidkhunthod P., Tang Y., Zhai W., Li L. (2024). Ultrasound-triggered
surface reconstruction with improved carriers transfer kinetics in
CdIn_2_S_4_ for solar water oxidation. Adv. Funct. Mater..

[57] Kim J. H., Jang Y. J., Kim J. H., Jang J.-W., Choi S. H., Lee J. S. (2015). Defective ZnFe_2_O_4_ nanorods with
oxygen vacancy for photoelectrochemical water splitting. Nanoscale.

[58] Xin Y., Cao X., Bao S., Ji S., Li R., Yang Y., Zhou H., Jin P. (2017). Two-step fabrication
of Na_x_WO_3_ thin film via oxygen-vacancy-induced
effect for energy
efficient applications. CrystEngComm.

[59] Zhang R., Ning F., Xu S., Zhou L., Shao M., Wei M. (2018). Oxygen vacancy engineering
of WO_3_ toward largely enhanced
photoelectrochemical water splitting. Electrochim.
Acta.

[60] Ng C., Iwase A., Ng Y. H., Amal R. (2013). Understanding self-photorechargeability
of WO_3_ for H_2_ generation without light illumination. ChemSusChem.

[61] Zhang Z., Tan X., Yu T., Jia L., Huang X. (2016). Time-dependent formation
of oxygen vacancies in black TiO_2_ nanotube arrays and the
effect on photoelectrocatalytic and photoelectrochemical properties. Int. J. Hydrog. Energy.

[62] Wang Z., Mao X., Chen P., Xiao M., Monny S. A., Wang S., Konarova M., Du A., Wang L. (2019). Understanding the roles
of oxygen vacancies in hematite-based photoelectrochemical processes. Angew. Chem..

[63] Corby S., Francas L., Kafizas A., Durrant J. R. (2020). Determining the
role of oxygen vacancies in the photoelectrocatalytic performance
of WO_3_ for water oxidation. Chem.
Sci..

[64] Yan L., Dong G., Huang X., Zhang Y., Bi Y. (2024). Unraveling
oxygen vacancy changes of WO_3_ photoanodes for promoting
oxygen evolution reaction. Appl. Catal. B: Environ..

[65] Yang M., He H., Du J., Peng H., Ke G., Zhou Y. (2019). Insight into
the kinetic influence of oxygen vacancies on the WO_3_ photoanodes
for solar water oxidation. J. Phys. Chem. Lett..

[66] Cheng S., Xiao Y., Yang S., Cai X., Gao Q., Yang X., Zhang S., Zhang S. (2025). Interfacial oxygen
acancy engineering in TiO_2_ nanowire/WO_3-x_ nanosheet
heterostructures for enhanced photoelectrochemical chemical oxygen
demand measurement. Sensors Actuat. B Chem..

