# Supplementary material for: Stable Solar Water Splitting Enabled in Anodic W/WO3 Nanorod Based Electrodes by Hydrothermal Engineering
Source: ACS Appl Nano Mater. 2025 Sep 22;8(39):18990–9000. doi: 10.1021/acsanm.5c03456 (PMC12501940; doi:10.1021/acsanm.5c03456)
Supplement: Supplementary file 1 [file an5c03456_si_001.pdf]

Supporting Information

Stable Solar Water Splitting Enabled in Anodic W/WO_3 Nanorod Based Electrodes By Hydrothermal Engineering

Piyali Chatterjee[1]*, Daniel Piecha[1,2], Mateusz Szczerba[1,2], Olga Chernyayeva[3], Łukasz Gondek[4], Tomasz Uchacz[1], Grzegorz D. Sulka[1]*

[1]*Faculty of Chemistry, Jagiellonian University, Gronostajowa 2, 30-387, Krakow, Poland*

[2]*Doctoral School of Exact and Natural Sciences, Jagiellonian University, Lojasiewicza 11, 30-348 Krakow, Poland*

[3]*Laboratory of Surface Analysis, Institute of Physical Chemistry, Polish Academy of Sciences, Kasprzaka 44/52, 01-224, Warsaw, Poland*

[4]*Faculty of Physics and Applied Computer Science, AGH University of Krakow, Av. Mickiewicza 30, 30-059, Krakow, Poland*

*Corresponding author. Email: piyali.chatterjee@uj.edu.pl, piyali93physics@gmail.com, sulka@chemia.uj.edu.pl.

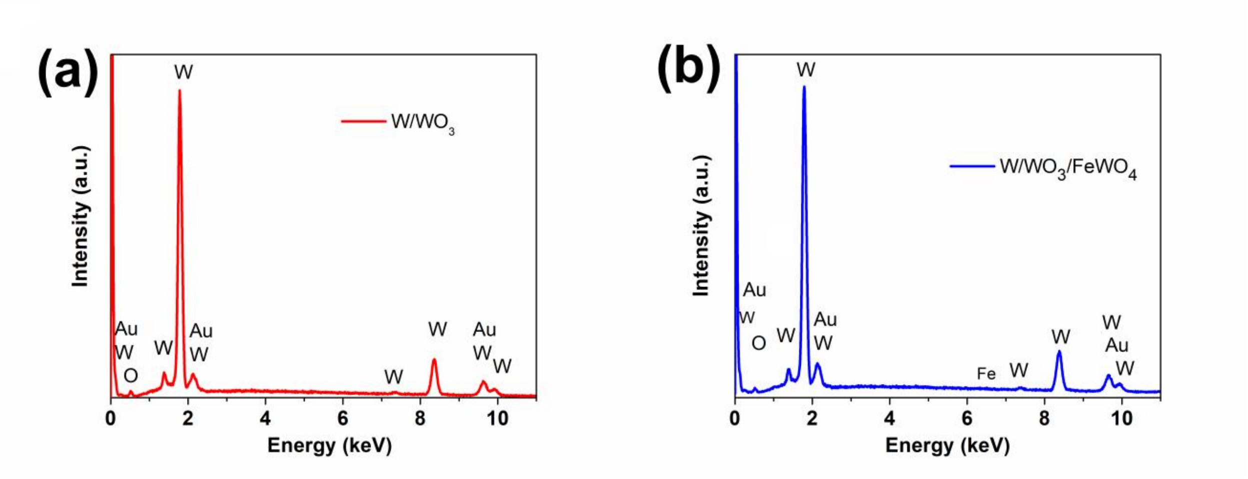

Figure S1. EDS spectra of (a) W/WO_3 and (b) $W/WO_3/FeWO_4$

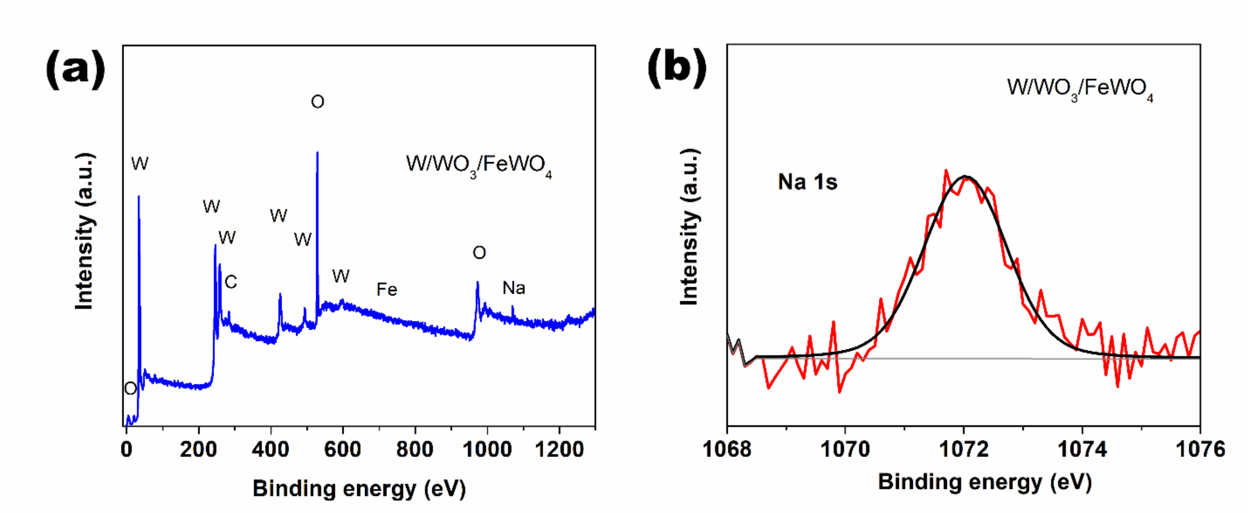

Figure S2. (a) XPS survey spectrum of the $W/WO_3/FeWO_4$ electrode. (b) High-resolution XPS spectrum of Na 1s for the $W/WO_3/FeWO_4$ electrode.

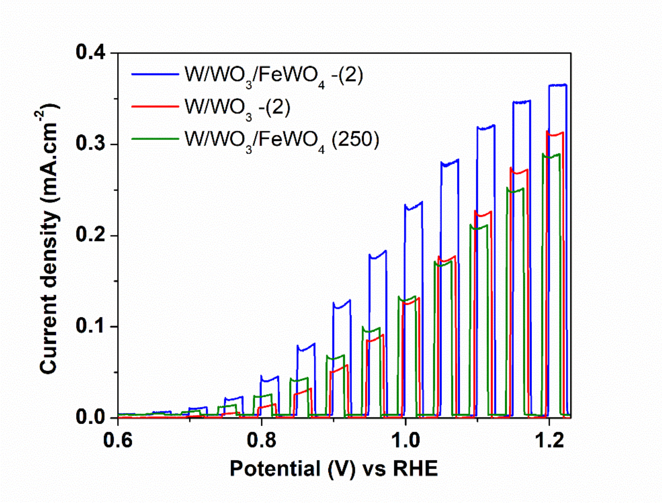

Figure S3. LSV curves of $W/WO_3/FeWO_4$ (250) compared to the best performing W/WO_3 and $W/WO_3/FeWO_4$ samples.

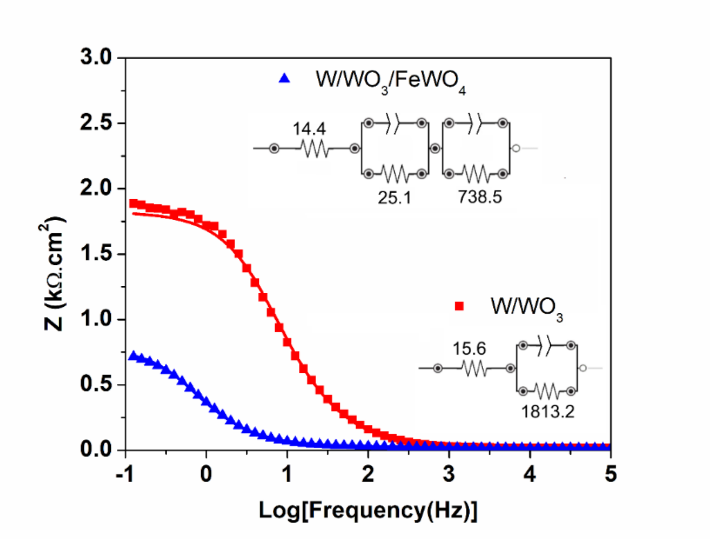

Figure S4: Bode diagram of W/WO_3 and $W/WO_3/FeWO_4$ under AM 1.5G illumination. Unit for values of resistive components in both the equivalent circuits is $\Omega.cm^2$.

Table S1. **Photoluminescence decay parameters for the studied materials recorded at 298 K, including lifetimes** (τ_1 , τ_2 , τ_3), **relative amplitudes** (A_1, A_2, A_3), and **averaged lifetimes** (τ_{av}).

Sample	λ_{em} [nm]	τ_1 [ns] (A_1)	τ_2 [ns] (A_2)	τ_3 [ns] (A_3)	τ_{av} [ns]	X^2
W/WO_3	460 nm	4.81 (0.12)	114.95 (0.50)	0.07 (0.38)	58.48	1.7
	515 nm	7.03 (0.18)	118.62 (0.74)	0.27 (0.08)	88.98	1.5
	580 nm	7.32 (0.21)	108.54 (0.71)	0.41 (0.08)	79.22	1.9
$W/WO_3/ FeWO_4$	460 nm	4.93 (0.11)	119.73 (0.50)	0.07 (0.39)	59.96	1.6
	515 nm	6.95 (0.16)	117.92 (0.73)	0.19 (0.09)	87.51	1.5
	580 nm	7.64 (0.20)	111.37 (0.71)	0.52 (0.09)	80.22	1.7

$\tau_{av,}$ the averaged photoluminescence lifetime was calculated using the following equation:
$\tau_{av} = \frac{\sum_1^n A_i \tau_i}{\sum_1^n A_i}$.

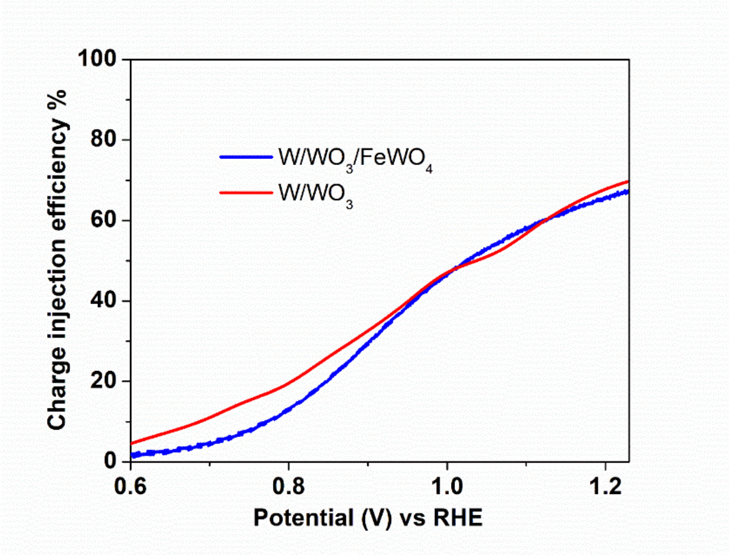

Figure S5. Charge injection efficiency in W/WO_3 and $W/WO_3/FeWO_4$ electrodes.

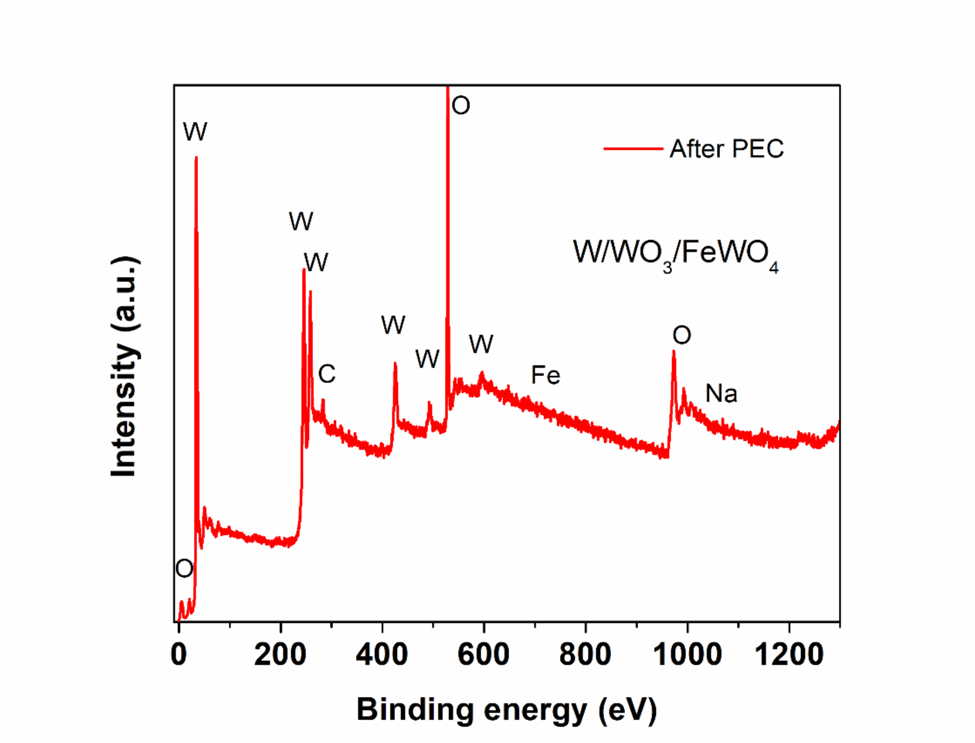

Figure S6. XPS survey spectrum of $W/WO_3/FeWO_4$ after the photoelectrochemical stability test.

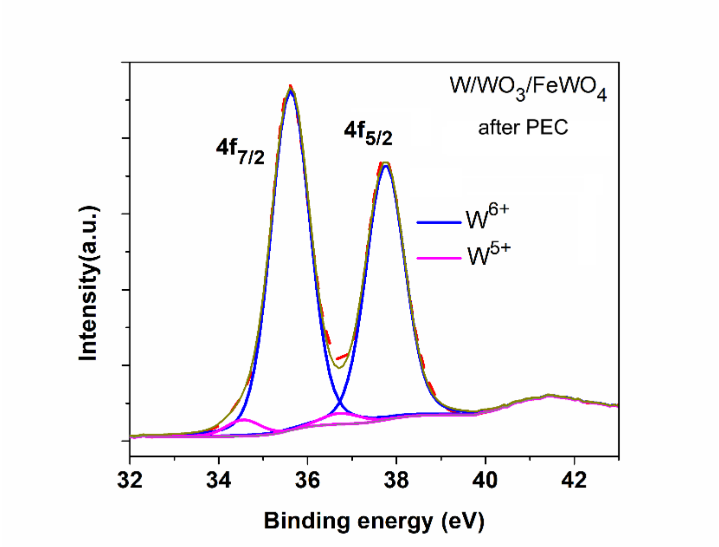

Figure S7. Deconvoluted high-resolution XPS spectrum of $W/WO_3/FeWO_4$ for W 4f after the stability test.

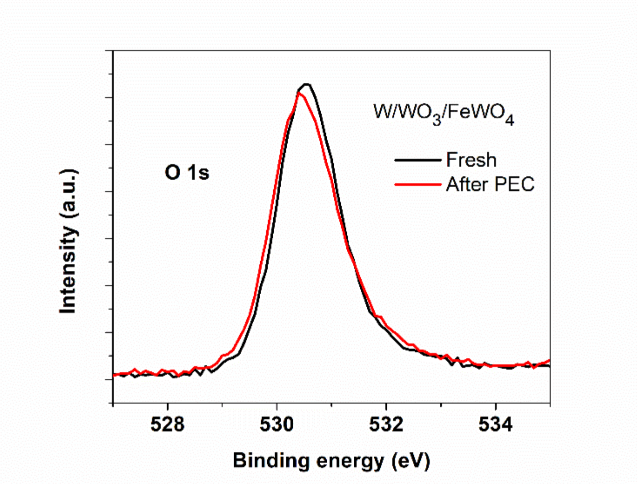

Figure S8. High-resolution XPS spectra of $W/WO_3/FeWO_4$ for O 1s before and after the stability test without sputter cleaning.

This research is part of the Project No. 2022/47/P/ST5/00813 co-funded by the National Science Center and the European Union Framework Program for Research and Innovation Horizon 2020 under the Marie Skłodowska-Curie grant agreement no. 945339.
